# Isolated Tubal Torsion in a Term Pregnancy: Case Report and Systematic Review of Literature of the Last 10 Years

**DOI:** 10.3389/fsurg.2022.856915

**Published:** 2022-04-05

**Authors:** Ferdinando Antonio Gulino, Carla Ettore, Gianfranco Morreale, Stefano Siringo, Emanuele Russo, Marco D'Asta, Francesco Cannone, Giuseppe Ettore

**Affiliations:** ^1^Medical Doctor, Department of Obstetrics and Gynaecology – Azienda di Rilievo Nazionale e di Alta Specializzazione (ARNAS) Garibaldi Nesima, Catania, Italy; ^2^Professor of Obstetrics and Gynaecology, Department of Obstetrics and Gynaecology – Azienda di Rilievo Nazionale e di Alta Specializzazione (ARNAS) Garibaldi Nesima, Catania, Italy

**Keywords:** isolated tubal torsion, acute abdominal pain, salpingectomy, ultrasound, pregnancy

## Abstract

**Objective:**

Isolated torsion of a fallopian tube is a rare event and it is extremely difficult to be diagnosed in pregnancy. The aim of this study is to present a clinical case report that occurred in our department and to summarize the latest evidence about tubal torsion in pregnancy.

**Methods:**

We reported data, ultrasonographic features and an intra-operative image of a case report of tubal torsion in a term pregnancy. Then a review of the literature was performed following the PRISMA statement: we searched all the articles related to tubal torsion in pregnancy in the last 10 years from the international electronic bibliographic database PUBMED. We collected data regarding population characteristics, clinical features, treatment, and feto-maternal outcomes.

**Results:**

According to our search strategy, 10 articles were included. The main clinical symptoms were abdominal pelvic pain (100%), nausea, and vomiting (30%). The mean gestational age at the diagnosis was 36 weeks after the last menstrual period in 50% of cases. Ultrasound images showed a cystic lesion in the adnexal area in 70% of cases. In most of the cases, a cesarean section with a contextual salpingectomy was performed. No cases of maternal and fetal death were respectively reported.

**Conclusion:**

Isolated torsion of the fallopian tube is a rare obstetric condition but it should be considered in case of acute lower abdominal pain presentation during pregnancy. Depending on gestational age, surgical treatment as soon as possible could prevent a salpingectomy.

## Introduction

Isolated torsion of a fallopian tube in pregnancy is a rare event in women of reproductive age (1). It could also occur in absence of an adnexal mass (2). This pathological condition has a differential diagnosis with all other causes of acute pelvic pain, both gynecological (such as hemorrhagic ovarian cyst, rupture of ovarian cyst, rupture of an endometriotic cyst or dermoid cyst, extra-uterine pregnancy, adnexal torsion, tube-ovarian abscess) (3), and general surgical causes (such as appendicitis, bowel obstruction, bowel perforation, peritonitis). The aim of this study is to present a clinical case report that occurred in our department and to summarize the latest evidence about tubal torsion in pregnancy.

## Materials and Methods

### Case Report

A 23-year-old primipara was admitted to our obstetric emergency unit at 38 weeks and 5 days of gestational age with acute abdominal pain. This pain was situated in the left lower abdomen, and it was constant and acute. She did not complain of nausea, vomiting, or diarrhea. There was no history of urinary symptoms, fever, or vaginal bleeding. The patient had a similar recurring pain for about 1 week, but she did not take any medication. Her anamnesis was uneventful, she had no medical pathology and she never had previous surgery. Until the occurrence of the pain, her pregnancy had been regular. The vital signs of the woman were stable: systolic blood pressure was 105 mmHg; diastolic blood pressure was 70 mmHg; heart rate was 103 bpm; SpO_2_ 98%; temperature 36.8°C / 98°F.

Abdominal examination revealed tenderness in the left lower abdomen and a soft abdomen. The uterine fundal height was correspondent to the period of gestation. Cardiotocography showed irregular uterine contractions, not perceived by the patient. The fetal heart rate was Category I (FIGO classification). The vaginal examination showed that the cervix was closed, Bishop index O, the fetal membranes were intact and there was not any evidence of bleeding or abnormal discharge.

An ultrasonographic scan showed a single alive fetus in cephalic presentation with corresponding biometry and a normal amniotic fluid index (AFI = 11 cm); the position and thickness of the placenta were normal, without any sign of abruption. An anechoic mass (65 mm × 46 mm) was observed in the left lower abdomen, with evidence of good posterior enhancement. There was no abnormal vascular flow on Doppler examination. The ovaries were not well assessed due to the uterine expansion of term pregnancy. There was no fluid in the pouch of Douglas. The laboratory blood parameters were as follows: 8.500/mm^3^ white blood cells (WBCs), 3.61 ×10,12/L red blood cells (RBCs), 12.0 g/dl hemoglobin (HGB), 1.56 reactive protein c (CRP), and 190.000 platelets (PLTs). The laboratory parameters for renal and hepatic function were normal.

**Figure 1:**
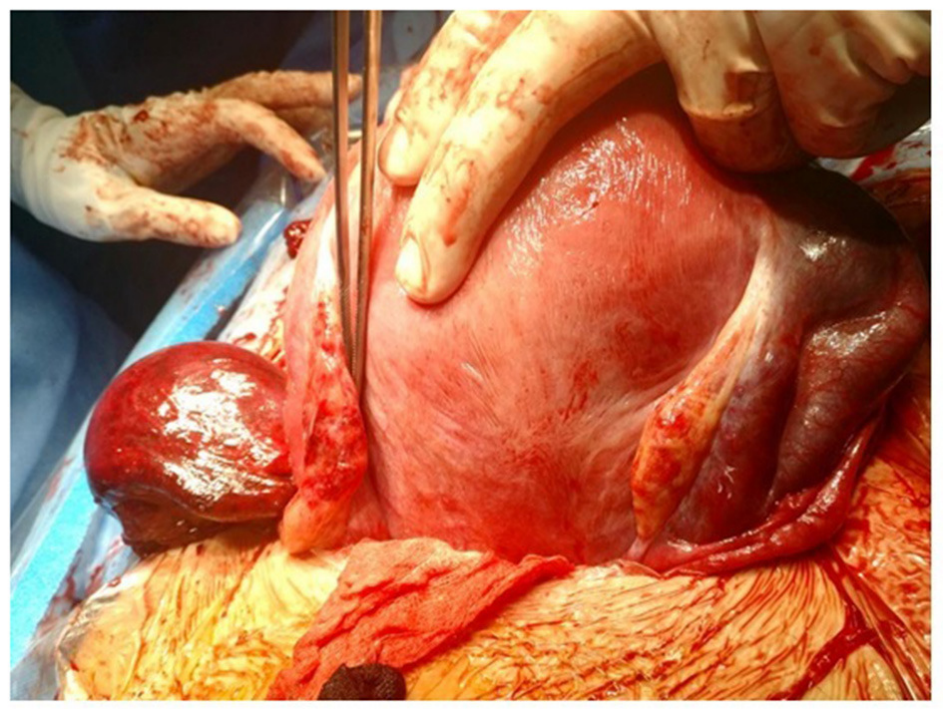
Triple isolated torsion of left Fallopian tube with healthy ovary.

The pregnant woman was hospitalized and the pain was unchanged after treatment with paracetamol 1 g every 6 h and phloroglucinol 80 mg. The onset of the pain was sudden and paracetamol was ineffective as pain relief. After hospitalization abdomen became tense. Due to her pregnancy status and the insufficient basis for a definitive diagnosis, a suspicion of a left ovarian cyst was considered, and it was decided to perform a surgical treatment. The patient underwent a cesarean section and an emergency exploratory laparotomy under spinal anesthesia. The findings were as follows: triple isolated torsion of the left fallopian tube had occurred and the tube presented edematous and purple, completely necrotic. The left ovary, the uterus, and the right adnexa looked healthy (Figure 1). After detorsion of the tube, it did not resume normal vascularization, so a left salpingectomy and cesarean section were performed.

The newborn was male, with a weight of 3,300 gr, an Apgar score of 10 at 1 min, and a normal score of 10 at 5 min. The patient was treated with intravenous antibiotics (cefazolin 1 g × 2/die) for 3 days and then it was continued for 2 days by intramuscular administration. The operation course and puerperium were uneventful, and the patient was discharged on the third postoperative day in good condition. The follow-up parameters of postpartum were all normal. The patient had no further complications. The histopathology report indicated that in the left fallopian tube there was hemorrhage and necrosis.

The study protocol was approved by the Ethics Committee of the ARNAS Garibaldi Hospital and conformed to the ethical guidelines of the Helsinki Declaration. The woman signed informed consent before entering the study, and their anonymity was preserved.

### Review of Literature

A review of the literature was performed following the PRISMA Statement (Preferred Reporting Items for Systematic Reviews and Meta-Analysis). In the last 10 years, we searched all the articles related to our topic from the international electronic bibliographic databases PUBMED (from 01/01/2011 to 31/03/2021). The articles were found using comprehensive search criteria and a combination of MeSH terms. We used the following words for selection: (“tube torsion” “salpingectomy and pregnancy” and “tubal torsion and pregnancy”). We selected the articles published between January 2011 and March 2021. The search was limited to studies reported in the English language. The references of the items chosen were also evaluated for related citations. Two independent researchers assessed the titles and abstracts retrieved to select the most relevant articles. The full text was obtained if the title and abstract did not provide enough information. Letters to editors, editorials, review articles, duplicates, meta-analysis were excluded. We only included items that followed our eligibility criteria, represented by pregnant women with a diagnosis of tubal torsion.

We excluded those studies evaluating data from women with ovarian torsion, adnexal torsion, patients not in pregnancy, and finally postmenopausal patients.

We also included articles concerning single case reports of tubal torsion where both treatment and the maternal prognosis were discussed. We collected data regarding population characteristics, clinical features, treatment, and feto-maternal outcomes.

## Results

According to our search strategy, 10 articles were included (4–13) (Table 1). We found 10 cases of isolated tubal torsion in pregnancy. The main clinical symptoms were abdominal pelvic pain (100%), nausea, and vomiting (30%). In 40% of cases, the patient reported a few days earlier a similar pain.

**Table 1:** Systematic research of the literature.

**Reference**	**Study design**	**Clinical features**	**Diagnostic imaging**	**Treatment**	**Findings**
Park (4)	Case report	35 + 6 weeks acute right abdominal pain that began 2 days prior to visit. direct tenderness without rebound tenderness was the only abnormal finding in her physical examination.	**US** showed a 37 mm sized hypoechoic cystic mass in the right lower quadrant. **TAC** shows a 44 × 25 mm sized homogenous cystic mass occupies the right lower quadrant	Cesarean section and right salpingectomy	Torsion of midportion of the right fallopian tube and necrosis measuring 6 × 4 cm size was observed. The cystic mass observed on CT scan and ultrasonography was hydrosalpinx which was thought to be the result of ischemic or traumatic tubal injury due to torsion
Bakacak and Bakacak (5)	Case report	37 weeks during labor sudden pain in the right lower abdomen	Preoperative diagnosis was considered as abruptio placenta.	Cesarean section and right salpingectomy	Isolated torsion of the right fallopian tube
Ergenoglu et al. (6)	Case report	20 weeks Acute abdominal pain	**US** paratubal cystic mass	Exploratory laparotomy and left salpingectomy	Paratubal cystic mass and left fallopian tube twisted among themselves. The fallopian tube was necrotic.
Işçi et al. (7)	Case report	Term pregnancy Acute abdominal pain	**US** normal findings	Cesarean section and salpingectomy	Fallopian tube torsion with findings of hemorrhagic infarct
Cate et al. (8)	Case report	35 + 4 weeks the onset of the pain was sudden and paracetamol was ineffective as pain relief. A few days earlier the patient had experienced a similar pain which had resolved spontaneous	**US** showed a cystic lesion of 4, 4 cm situated on the left side lateral of the uterus. **MRI** T2-weighted images showed a segmental dilation of the distal left tuba in proximity of the normal appearing ipsilateral ovary. The radiologic diagnosis of an isolated fallopian tubal torsion or torsion of a (para)tubal cyst was suggested	Labor was induced with vaginal application of Dinoproston. After 12 h a cesarean section was performed because of persistent pain, unresponsive to tramadol, in absence of cervical dilatation. Detorsion surgery was able to prevent the need for salpingectomy.	Edematous and purple left fallopian tube. A 3 fold torsion around its long axis was observed. The tubal fimbriae were also purple and enlarged
Macedo et al. (9)	Case report	33 weeks generalized abdominal pain. She reported a 1-day history of generalized abdominal pain, nausea, and vomiting.	**US** of the abdomen could not visualize the appendix **MRI** results were inconclusive, revealing a fluid collection in the right lower quadrant, but without definitive appendicitis. The radiologist hypothesized ruptured appendix, ruptured ovarian cyst, or peritoneal inclusion cyst as possible sources of the fluid	Diagnostic Laparoscopy: appendicectomy and right salpingectomy	In surgery a necrotic fluid filled mass was noted in the right lower quadrant. The structure was revealed to be a torsed fallopian tube without ovarian involvement. Inspection of the appendix revealed a sclerotic distal third appendix. Pathology of the appendix tissue revealed localized inflammation suggested an early developing appendicitis.
Simsek et al. (10)	Case report	36 weeks Acute pain in right lower quadrant that emanating to right lomber region	**US** showed grade 3 hydronephrosis in right kidney	Cesarean section + right salpingectomy	Twisted right tube. Right ovary was normal in appearance. The torsioned tube compressed the right ureter and caused ureteral dilatation
Sun et al. (11)	Case report and review	36 + 4 weeks Right lower abdominal pain for 3 days	**US** anechoic mass in the right lower abdomen, with evidence of good posterior enhancement, no abnormal vascular flow on Doppler examination with suspicion of appendicitis	Emergency exploratory laparotomy: cesarean section and right salpingectomy	Isolated 360° torsion of the right fallopian tube had occurred at the proximal end of the isthmus, and part of the right tube from the isthmus to the fimbriae showed complete infarction
Duncan et al. (12)	Case report	30 + 3 weeks sudden onset severe right-sided abdominal pain described as an intermittent sharp stabbing pain radiating to her right flank. Her pain was associated with three episodes of vomiting.	**US** 47 mm oblong cyst was seen in the right adnexa	Exploratory laparoscopy: right salpingectomy	A torted right hydrosalpinx was seen with an associated fimbrial cyst. The cyst was approximately 50 mm and haemorrhagic but was otherwise simple in appearance. The torted hydrosalpinx was detorted; however, the fallopian tube had been compromised
Chohan et al. (13)	Case report	35 + 4 weeks acute-onset right lower quadrant pain 8/10, nausea and vomiting, and uterine contractions.	**US** 9.2 × 5.8 × 4.2-cm right adnexal cyst, 3.1 × 1.6 × 2.1-cm right ovary, and normal Doppler flow	Laparoscopic drainage of a paratubal cyst, with untwisting of the fallopian tube	A normal appearing right ovary, a large right paratubal cyst and the right fallopian tube twisted 360 degrees

The mean gestational age at the diagnosis was 36 weeks after the last menstrual period in 50% of cases; in a term pregnancy in 20% of cases (in one case during labor); in an early preterm (30–33 weeks) pregnancy with a rate of 20%; finally, it occurred in the second trimester in 10% of cases. Ultrasound images showed a cystic lesion in the adnexal area in 70% of cases. This was not diagnostic but raised the suspicion of a gynecological cause of acute pain. Magnetic Resonance Imaging (MRI) was performed in two cases but its findings could characterize the cystic lesion and suggested the right diagnosis only in one case. In a single case, abdominopelvic computed tomography was carried out and it showed a homogenous cystic mass in the lower quadrant; therefore, it did not add anything to ultrasound findings (4).

After 36 weeks of gestation tubal torsion was diagnosed during cesarean section with clinical suspicion (60%), in one case it was treated by laparoscopy. In early preterm pregnancies (30–33 weeks) it was diagnosed by diagnostic laparoscopy, in one case by a laparotomic exploration (6).

Surgical findings were an edematous and purple fallopian tube, with torsion around its long axis and complete infarction, so it needed salpingectomy in 80% of cases.

In one case inspection of the appendix revealed a sclerotic distal third appendix. The histologic study of the appendix tissue revealed localized inflammation, suggesting an early developing appendicitis (9). In only one case a normal position of the fallopian tube was obtained by detorsion, and quick revascularization occurred (8). In another case, the torsion of the tube compressed the right ureter and caused ureteral dilatation (10). No cases of maternal and fetal death were respectively reported.

## Discussion

Torsion of the fallopian tube is an uncommon condition in pregnant women and it is not often reported in scientific literature. It has to be differentiated with many obstetrical conditions, such as extra-uterine pregnancy, abruptio placentae or uterine rupture; gynecological conditions, such as hemorrhagic ovarian cyst, rupture of ovarian cyst, rupture of an endometriotic cyst or dermoid cyst, adnexal torsion, tube-ovarian abscess, torsion or degeneration of a leiomyoma; and, finally, with the general surgical condition, such as acute appendicitis, bowel obstruction, bowel perforation, peritonitis, cholecystitis, ureteral and renal colic. This wide range of pathological conditions makes the diagnosis extremely difficult, particularly on pregnant women in the third trimester. The use of abdominopelvic computed tomography has to be avoided in pregnancy, therefore the main diagnostic instrument to diagnose this condition is represented by ultrasound, but in a term pregnant patient, it is not easy to have a clear evaluation of the adnexa, due to uterine expansion. In addition, most of the clinical symptoms may be confused by the presence of a concomitant, or consequent term or preterm labor.

A previous review on this field was performed and published in 2009 by Origoni et al. (14): they described only 19 cases in literature from 1936 to that time. They conclude that the main predisposing factors for isolated fallopian tube torsion in pregnancy are para-ovarian cysts such as Morgagni hydatids, hydrosalpinges, and ovarian cysts, so they suggest excising pedunculated and non-pedunculated hydatids of Morgagni and para-tubal cysts when incidentally found during abdominal and pelvic surgical procedures. They also consider laparoscopy as the best treatment option until 32–34 weeks of gestation.

Another aspect to consider is the anesthesia for this surgical procedure: it depends mainly on weeks of gestation (term or preterm pregnancy) and on the type of procedure (laparoscopy or laparotomy). Some authors have described previously a case of laparoscopic removal of a perforated intrauterine device (IUD) during the first trimester of pregnancy under regional anesthesia (15) as a feasible and safe option that could be considered when needed, but current evidence (16) suggests no significant advantages to using spinal anesthesia over general anesthesia for laparoscopic treatment of gynecological diseases outside of pregnancy.

In our review of the last 10 years, we found 10 cases, and in most of them the main problem to solve was always the diagnosis; in most of these cases, the diagnosis was performed surgically, by laparoscopy, or, more frequently by laparotomy, in a contextual cesarean section procedure. We agree with Origoni et al. (14) that preoperative detailed Doppler flow ultrasound evaluation of the uterine adnexa could be predictive for the diagnosis of isolated tubal torsion when a cystic para-ovarian structure is identified in the pelvis and the ipsilateral ovary appears normal; however, in our case report, but also in two cases described in our review, there was no abnormal vascular flow on Doppler examination.

## Conclusion

Isolated torsion of the fallopian tube is very rare, is an infrequent obstetric condition but should consider in case of acute lower abdominal pain presentation during pregnancy. Considering that this sign is not specific, the cases are frequently misdiagnosed as having other causes of acute abdominal pain. Ultrasound images are not diagnostic but could help in the differential diagnosis.

Cesarean section is performed in most of the cases with contextual salpingectomy, considering the gestational age at diagnosis. Depending on gestational age, surgical treatment as soon as possible could prevent a salpingectomy.

## Author Contributions

FG and FC conceived the project of this work and designed the experiment. CE and GM made the clinical activity and following the patient in their appointments. SS made the review of literature. GE was the clinical and scientific coordinator of the study. All authors contributed to the article and approved the submitted version.

## Conflict of Interest

The authors declare that the research was conducted in the absence of any commercial or financial relationships that could be construed as a potential conflict of interest.

## Publisher's Note

All claims expressed in this article are solely those of the authors and do not necessarily represent those of their affiliated organizations, or those of the publisher, the editors and the reviewers. Any product that may be evaluated in this article, or claim that may be made by its manufacturer, is not guaranteed or endorsed by the publisher.
